# Differentiation and response mechanisms of the endophytic flora of plants ecologically restored in the ilmenite area

**DOI:** 10.3389/fmicb.2025.1555309

**Published:** 2025-03-07

**Authors:** Xin Yu, Junqiang Xu, Ziping Zou, Yunfeng Zhang, Peng Wu, Qiang Li

**Affiliations:** ^1^Key Laboratory of Coarse Cereal Processing, Ministry of Agriculture and Rural Affairs, Sichuan Engineering and Technology Research Center of Coarse Cereal Industrialization, School of Food and Biological Engineering, Chengdu University, Chengdu, China; ^2^Yunnan Plateau Characteristic Agricultural Industry Research Institute, Yunnan Agricultural University, Kunming, China

**Keywords:** soil pollution, rhizosphere soil, endophytic bacteria, heavy metals, ecological restoration

## Abstract

Heavy metal contamination in soil is a serious environmental challenge, and abandoned mining areas are of particular concern. In order to rehabilitate the ecology of these areas. In this study, we used ICP-MS and potentiometric method to analyze the soil physicochemical and then endophytic bacteria of remediation plants with the help of 16sRNA sequencing, in order to investigate the ecological remediation of abandoned ilmenite mine and the effect of soil microbiology by seven common plants. The results revealed that the abandonment of ilmenite significantly increased the contents of total phosphorus, total potassium, available potassium, iron, and lead in the surrounding soils. It also affected the richness and diversity of endophytic bacterial communities. Pvi had the highest richness, while Tsi had the lowest richness (*P* < 0.05). A total of 28 phyla, 69 classes, 171 orders, and 521 genera were identified. A total of nine core OTUs were found: *Stenotrophomonas*, *Chryseobacterium*, *Lactobacillus*, *Clostridium_sensu_stricto_12*, *Prevotella*, *Lactobacillus*, *Bradyrhizobium*, *Nocardioides*, and *Delftia*. Beta diversity analysis revealed that the community structure of the endophytic bacteria differed during the remediation process at the ilmenite site. Functional prediction revealed upregulation of Dco transporter protein function, DNA-binding transcriptional regulators, glyoxalase or related metal-dependent hydrolases, acyl coenzyme A synthetases, ATPase components, amino acid synthesis, and cellular respiration-related functions. Pearson correlation analysis revealed that the SOC, TK, AN, AK, and Zn contents were significantly correlated with α diversity. Redundancy analysis (RDA) revealed that Actinobacteriota was significantly positively correlated with soil SOD, AN, TN, and TK contents. For the first time, this study revealed the interactions among plants, endophytic bacteria and soil pollutants, laying a theoretical basis for screening specific plant endophytic bacteria for ecological restoration.

## 1 Introduction

Heavy metal-contaminated soil is a severe problem in most countries. In recent decades, to promote regional economic growth, mineral mining has led to an increase in metal pollution in the environment (Zhong et al., 2023; Su et al., 2022). Heavy metals have polluted one-sixth of the agricultural land in China. With the continuous expansion of infrastructure construction, the demand in the steel, high-speed rail, aerospace, and chemical industries has increased significantly (Dushyantha et al., 2020). Ilmenite mining is also active. However, with ore mining, stone coal smelting, and fossil fuel combustion, large amounts of waste liquid and residues enter the soil (Xiao et al., 2017). The processing of 1 ton of iron concentrate will generate approximately 1.5 tons of vanadium-titanium-magnetite tailings, which pose a continuous threat to terrestrial ecosystems and health risks to local residents (Liu et al., 2019). Previous studies have reported that the titanium content in tailings accounts for 6.46% of the total titanium content (Zhai et al., 2020). Under the action of atmospheric precipitation and rainwater erosion, the levels of As, Cd, and Pb in the plants around the mining area exceeded the allowable limits (Vejvodová et al., 2022). In the past 20 years, the ecological restoration of heavy metals has mainly used physical (Liu et al., 2024a; Jiang et al., 2024) and chemical methods (Wu et al., 2022; Zhang et al., 2016; Zhang et al., 2024; Chang et al., 2018); however, these methods all have the disadvantages of high cost, high labor intensity, and potential secondary impacts on the environment. Hence, it is imperative to investigate restoration methods that are both cost-effective and eco-friendly.

Contaminated environments contain an abundance of microbes that can adapt to extreme conditions (Liu et al., 2022a), and the study of these microorganisms can increase our understanding of microbial diversity (Liu et al., 2022b); At the same time, the study of the biodiversity of endophytic bacteria and the physicochemical properties of soil contaminated by heavy metals is critical for environmental protection and restoration of the ecological damage caused by mining activities. However, the above studies have not yet determined the impacts on local plants and microbes during the ecological restoration process.

The growth and quality of crops are heavily influenced by bacteria. By impacting the creation of plant hormones, improving soil nutrient availability, strengthening plant disease resistance, and decomposing organic material, bacteria can elevate crop yield and enhance crop quality (Chebotar et al., 2015; Jayakumar et al., 2019; Hao et al., 2024). As a type of bacteria, endophytic bacteria also play a similar role (Afzal et al., 2019). Endophytic bacteria-mediated stress tolerance is considered to be the most successful technique for bioremediation because this is environmentally acceptable, economically sound, and technically feasible (Singh et al., 2015). When plant growth is stressed, endophytic bacteria have a more beneficial effect on host plants than rhizosphere bacteria do (Hardoim et al., 2008). Endophytic bacteria not only eliminate competition problems (Chatterjee et al., 2010), but can also withstand very high concentrations of heavy metals. In addition to making siderophore and inorganic phosphate, it solubilizes and mobilizes heavy metal (Chen et al., 2010). Endophytic bacteria may interact more closely with their hosts than inter-root bacteria. Plants provide nutrients and a habitat for bacteria, which may directly or indirectly improve plant growth and health (Mastretta et al., 2006). Endophytic bacteria can indirectly benefit plant growth by preventing the growth or activity of plant pathogens through competition for space and nutrients, production of hydrolytic enzymes, antimicrobials, induction of plant defense mechanisms, and inhibition of enzymes or toxins produced by pathogens (Weyens et al., 2009). Previous studies have shown that endophytes are potential sources of highly efficient biosorbents for biosorption of heavy metals (Xiao et al., 2010). Endophytic bacteria help plants cope with metal stress by converting metal ions to less toxic or nontoxic forms, thereby reducing the toxicity of heavy metals (Zhu et al., 2014). However, the impact of heavy metal pollutants on the composition and activity of the endophytic bacterial community remains unclear. Furthermore, the ways in which different plants react to metal pollutants and their functional variations are not yet fully understood. Therefore, we performed the following study.

This study used 16S rRNA sequencing technology on the Illumina NovaSeq platform to investigate the effects of ecological restoration on the diversity and community structure of endophytic bacteria in the ilmenite area. We selected seven species of plants with strong environmental adaptability that were planted by our team for the ecological restoration of the mining area 2 years ago as samples to explore the ecological restoration effect of ilmenite control. This study documents the impacts of ecological restoration in the ilmenite area on the structure and function of plant endophytic bacterial communities, as well as the response mechanisms to the differentiation of endophytic bacterial communities and functional differentiation. This research offers a theoretical framework for a thorough comprehension of how ilmenite ecological restoration affects soil microecology and environmental restoration. It also sets the groundwork for identifying valuable microbial resources.

## 2 Research area and methods

### 2.1 Study locations and sample collection

The samples for this study were collected in May 2024. Seven environmentally adapted plants were planted by our team in June 2022 at an abandoned mine site (within 3 kilometers of the ilmenite area) in Wuding County, Chuxiong Yi Autonomous Prefecture, Yunnan Province: *Polygonum plebeium* R. Br., *Tournefortia sibirica* L., *Alhagi camelorum* Fisch., *Casuarina equisetifolia* L., *Dryopteris coreano-montana* Nakai, *Dodonaea viscosa* Jacquem., and *Pteris vittata* L. These plants were specifically named Ppl, Tsi, Aca, Ceq, Dco, Dvi, and Pvi (Supplementary Table S1). Three groups of replicates were selected as soil samples for each plant. The sampling methods were performed according to the method described by Li et al. (2022). The plant surfaces were disinfected with alcohol before they were placed in sterile bags and placed in an incubator filled with ice packs for temporary storage. All collected samples were transported to the laboratory under refrigeration for DNA extraction and rDNA sequencing. First, a DNA kit (MP Bimonedicals, United States) was used for extraction, and the extraction effect was detected on a 1% agarose gel.

### 2.2 Measurement of soil physicochemical properties

Soil pH was determined using the potentiometric method (HJ 962–2018), and organic carbon (SOC) was determined using fast microwave digestion (Benbi, 2018). The total nitrogen (TN) was determined via the Kjeldahl method (Mason et al., 1999), the total phosphorus (TP) was determined through nitric acid wet digestion (Webb and Adeloju, 2013), the total potassium (TK) was determined using atomic absorption spectrometry (Miswan et al., 2023), then alkaline nitrogen (AN) was determined using the alkaline hydrolysis diffusion method (Tsiknia et al., 2014), the available phosphorus (AP) and available potassium (AK) were determined using infrared spectroscopy (Jia et al., 2015). The contents of five heavy metals in the soil, including iron (Fe), zinc (Zn), copper (Cu), titanium (Ti), and lead (Pb), were determined using ICP-MS (Moor et al., 2001).

### 2.3 PCR amplification detection

The extracted genomic DNA was diluted to 1 ng/μL with sterile water and incubated with conventional primers (799F, 5′-AACMGGATTAGATACCCKG-3′; 1193R: 5′-ACGTCACCCTTCC-3′) for the 16S rRNA V5-V7 region of all samples. In this step, 15 μL of Phusion® High-Fidelity PCR Master Mix (New England Biolabs), 2 μL of forward and reverse primers and 10 ng of template DNA were used. Thermal cycling conditions were performed according to the method of Li et al. (2022) and finally electrophoresed on agarose gels. Next, the extract was purified using a Qiagen Gel Extraction Kit (Qiagen, Germany).

### 2.4 Library preparation, sequencing, and data processing

One microliter of the library was collected, and quality control was performed on the library using the Agilent High Sensitivity DNA Kit. Library quantification was performed using a Quant-iT PicoGreen dsDNA Assay Kit from Promega QuantiFluor. Qualified libraries were subjected to paired-end sequencing on the Illumina NovaSeq instrument (Modi et al., 2021; Wu et al., 2021).

### 2.5 OTU clustering and species annotation

We assigned sequences with a similarity of ≥97% to the same operational taxonomic unit (OTU) using Uparse v7.0.1001 (Edgar, 2013) which were then filtered for representative sequences from each OTU and annotated using the Silva database (Quast et al., 2013). In order to investigate the phylogenetic relationship between OTUs and the variations in dominant species among different samples (groups), we conducted multiple sequence alignment using Muscle v3.8.3 (Edgar, 2004). Finally, the analysis of alpha diversity and beta diversity was performed using normalized data.

### 2.6 Data analysis

#### 2.6.1 Microbial alpha diversity analysis

By employing QIIME2 software, we calculated seven diversity indices for each sample (Hall and Beiko, 2018; Yang et al., 2024), which included the Chao1, Observed_otus, Shannon, Simpson, dominance, Pielou_e, and Good’s coverage. Following this, a box plot was created to compare the richness and uniformity of OTUs among different samples. For the observed species, Chao1 was used to identify the richness of the community. The Shannon and Simpson were used to identify symbiont diversity. Good’s coverage was used to describe the sequencing depth. Dominance and Pielou_e were used to describe the species evenness of the community.

#### 2.6.2 Microbial beta diversity analysis

Beta diversity is frequently employed to assess the variations in species diversity among samples. The beta diversity of the weighted UniFrac was calculated using QIIME 2 (Caporaso et al., 2010) along with a non-metric multidimensional scaling analysis using the R-VEGAN software package.

### 2.7 Functional prediction

In order to speculate on the function of endophytic bacteria, we employed Phylogenetic Investigation of Communities by Reconstruction of Unobserved States (PICRUST) (Douglas et al., 2018) and used the gene ontology (GO)(Ashburner et al., 2000) and Kyoto Encyclopedia of Genes and Genomes (KEGG)(Kanehisa and Goto, 2000). The function for cluster analysis first performs principal component analysis (PCA) using the FactoMineR program package and the ggplot2 package in R v2.15.3 (Almeida et al., 2018; Gould, 2019). Dimensionality reduction is then performed on the original variables.

### 2.8 Statistical analysis

All of the samples included three replicates. Analysis of variance (ANOVA) was performed using SPSS 21.0. *p* < 0.05 was considered significant.

## 3 Results and analysis

### 3.1 Soil element contents and canonical correlation analysis

The constant element analysis revealed that the nitrogen content in alkaline hydrolysis was the highest at 80.00 g/100 g. This was followed by organic carbon, available phosphorus, total phosphorus, total nitrogen, and total phosphorus (Table 1) (*p* < 0.05). The pH of Ceq was the highest at 8.02, and the pH of Ppl was the lowest at 6.93. Compared to those of the samples from the non-mining area (CK), only the pH values of Ppl and Pvi decreased, while those of the remaining samples significantly increased. In terms of organic carbon content, the levels of seven samples were markedly lower compared to the CK sample. The Aca content was the lowest at 1.95 g/100 g, while the Ceq content was the highest at 27.08 g/100 g. For the total nitrogen content, Ceq was the highest at 2.26 g/100 g; Ppl was the lowest at 0.29 g/100 g; Ceq, Dvi and Dco were greater than those of CK; and the contents of the remaining samples were lower than those of CK. In terms of total phosphorus, compared to CK, Ceq had the highest amount (0.21 g/100 g), while Pvi had the lowest (0.09 g/100 g) (*p* < 0.05). Except for Pvi, all other samples had higher amounts than CK. For alkaline-hydrolyzed nitrogen, we found that the contents of all samples were lower than those of the CK. Among the seven samples, Dvi had the highest value (165.00 g/100 g), while Ppl had the lowest (14.00 g/100 g) (*p* < 0.05). Analysis of the indicators for available phosphorus revealed that, compared to all the samples, the DVI content of the CK samples was the highest at 15.33 g/100 g, and the PVI content was the lowest at 2.83 g/100 g. Physicochemical examination revealed that the available potassium content of all the samples was significantly greater than that of CK, with Tsi having the highest content (367.00 g/100 g) and Dco having the lowest content (187.67 g/100 g) (*p* < 0.05).

**Table 1:** Soil physical and chemical properties.

Element (g/kg dw)	CK	Ppl	Tsi	Aca	Ceq	Dco	Dvi	Pvi
pH	7.15 ± 0.01 d	6.93 ± 0.18 f	7.21 ± 0.02 d	7.22 ± 0.03 d	8.02 ± 0.01 a	7.40 ± 0.03 c	7.02 ± 0.02 e	7.77 ± 0.01 b
SOC	28.16 ± 0.11 a	2.30 ± 0.05 f	2.65 ± 0.04 f	1.95 ± 0.02 f	27.08 ± 0.13 b	26.96 ± 0.50 b	4.51 ± 0.10 c	26.28 ± 0.49 b
TN	1.89 ± 0.01 c	0.29 ± 0.01 g	0.45 ± 0.01 e	0.37 ± 0.01f	2.26 ± 0.02a	2.18 ± 0.01b	0.76 ± 0.00 d	2.24 ± 0.21 a
TP	0.10 ± 0.00 g	0.17 ± 0.00c	0.18 ± 0.00 b	0.14 ± 0.00 e	0.21 ± 0.00 a	0.16 ± 0.00 d	0.09 ± 0.00 h	0.13 ± 0.00 f
TK	15.10 ± 0.12 e	14.20 ± 0.12 f	14.30 ± 0.12 f	15.13 ± 0.03 e	20.63 ± 0.15 c	22.87 ± 0.12 a	21.60 ± 0.10 b	19.27 ± 0.12 d
AN	218.00 ± 0.58 a	14.00 ± 0.58 g	18.00 ± 0.58 f	12.67 ± 0.88 g	157.00 ± 0.58 c	152.00 ± 1.15 d	44.33 ± 0.33 e	165.00 ± 1.53 b
AP	21.47 ± 0.50 a	5.80 ± 0.40 e	4.93 ± 0.24 e	3.83 ± 0.18 f	9.13 ± 0.38 c	5.43 ± 0.23 e	2.83 ± 0.19 f	15.33 ± 0.57 b
AK	144.67 ± 1.76 f	281.67 ± 1.45 b	367.00 ± 2.52 a	286.33 ± 2.03 b	251.67 ± 1.86 c	187.67 ± 0.88 e	238.33 ± 2.60 d	281.00 ± 3.06 b

In the detection of trace elements (Table 2), the maximum content of copper was 145 mg/kg, followed by zinc, iron, titanium, and lead. The copper content is an indicator. Except for Dco and Pvi, the rhizosphere soil of the remaining plants was greater than that of CK, with the highest rhizosphere soil content of Ceq at 319.00 ± 5.51 mg/kg and the lowest rhizosphere soil content of Pvi at 48.33 ± 0.67 mg/kg (*p* < 0.05). The Zn contents of the seven plant species were lower than those of CK, with Tsi having the highest content in rhizosphere soil at 96.67 ± 2.60 mg/kg, P in the rhizosphere soil had the lowest content at 30.33 ± 0.33 mg/kg. The iron content of all the samples was greater than that of the CK samples. The Ceq rhizosphere soil had the highest iron content, at 135.37 ± 3.98 mg/kg, while the Pvi rhizosphere soil had the lowest iron content, at 81.23 ± 1.91 mg/kg (*p* < 0.05). The titanium content of CK was the highest at 14.57 ± 0.34 mg/kg, and the titanium contents of the soil samples were as follows: Ppl, Tsi, Dco, Dvi, Pvi, Ceq, and Aca. In terms of Pb content, the Ppl rhizosphere soil had the highest content at 14.57 ± 0.34 mg/kg; the Ceq rhizosphere soil had the lowest content at 4.23 ± 0.03 mg/kg (*p* < 0.05).

**Table 2:** Soil trace element content.

Element (mg/kg dw)	CK	Ppl	Tsi	Aca	Ceq	Dco	Pvi	Dvi
Fe	73.47 ± 1.27 e	94.03 ± 0.29 b	91.80 ± 2.62 bc	85.00 ± 2.31 cd	135.37 ± 3.98 a	81.93 ± 1.80 d	81.23 ± 1.91 d	95.00 ± 2.84 b
Zn	109.33 ± 1.20 a	89.67 ± 2.03 c	96.67 ± 2.60 b	83.00 ± 2.31 c	81.67 ± 2.40 e	48.67 ± 0.88 g	30.33 ± 0.33 h	65.33 ± 1.86 f
Cu	112.33 ± 1.86 d	130.00 ± 3.51 c	137.67 ± 2.40 c	152.67 ± 2.40 b	319.00 ± 5.51 a	105.67 ± 2.40 d	48.33 ± 0.67 e	154.33 ± 4.10 b
Ti	14.57 ± 0.34 a	13.63 ± 0.37 b	12.00 ± 0.12 c	4.23 ± 0.03 g	4.80 ± 0.10 g	7.00 ± 0.15 e	5.60 ± 0.12 f	5.83 ± 0.03 f
Pb	5.83 ± 0.03 f	14.57 ± 0.34 a	13.63 ± 0.37 b	12.00 ± 0.12 c	4.23 ± 0.03 g	4.80 ± 0.10 g	7.00 ± 0.15 e	5.60 ± 0.12 f

In summary, the seven local plants used to control tailings pollution targeted Zn and Ti pollution and played an important role. Among these plants, Pvi had the most significant effect on the Zn control process. In the governance process of Ti, the effects of Aca and Ceq are the most significant. For the control of Cu pollution in tailings, Pvi could be degraded, and the effect was more significant (*p* < 0.05).

### 3.2 Sequencing data analysis

We detected the species richness of the endophytic bacteria of seven kinds of plants planted in the mining area: Ppl, Tsi, Aca, Ceq, Dco, Dvi, and Pvi. The sparseness curves of the OTU sample species are shown in Supplementary Figure S1. The sparse curve gradually stabilized when the number of sequencing reads reached more than 14,000, indicating that the sequencing results tended to be saturated. After removing chimeras, poor quality reads, and short reads, a 97% similarity threshold was used to assign OTUs. The number of OTUs per sample ranged from a minimum of 111 to a maximum of 1,034, with an average of 528 OTUs across all samples.

### 3.3 Classification and abundance of samples

Among all the samples, we identified a total of 28 phyla. There are 69 classes, 171 orders, and 521 genera. *Proteobacteria* accounted for 67.89%, *Actinomycetes* accounted for 24.65%, *Cyanobacteria* accounted for 2.89%, and *Firmicutes* accounted for 1.11%. Among the *Proteobacteria*, *γ-Proteobacteria* dominated (89.75%), and *Actinomycetes* also accounted for the highest proportion (77.92%) of the *Actinomycetes* phylum. At the genus level, *Steotrophomonas* had the highest abundance (58.15%), followed by *Streptavidin* (4.46%) and *Pseudonocardia* (3.20%).

### 3.4 Alpha diversity

**Figure 1:**
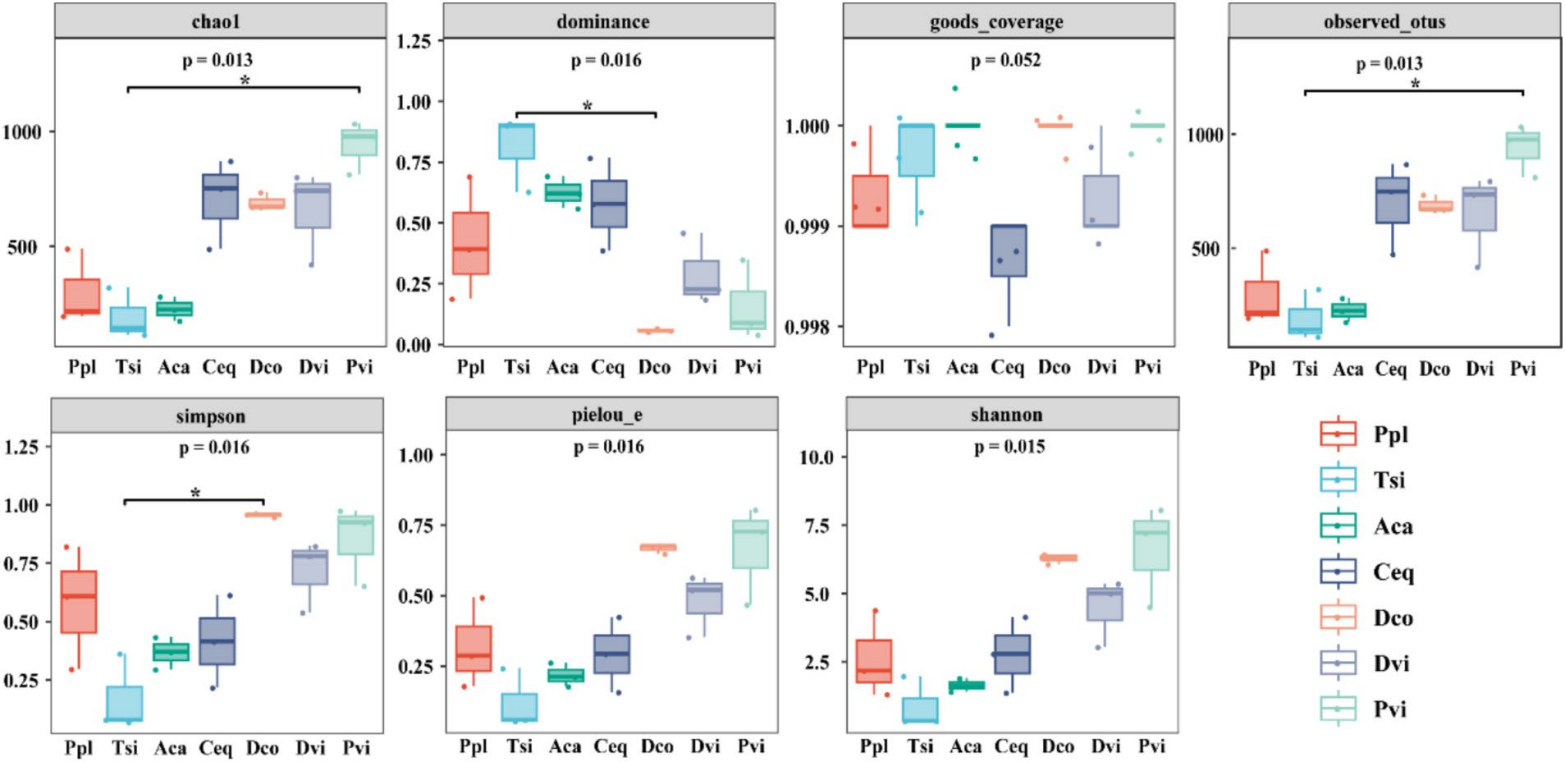
Changes in the alpha diversity indicators of different samples.

The diversity and complexity of the sampled species were revealed through seven indices. The diversity and complexity of the sampled species were revealed through seven indices. The data presented in Figure 1 indicates that as Alpha diversity increases, the diversity level of endophytic bacteria in the root system also increases. According to the community richness analysis, Pvi had the highest species richness, while Tsi had the lowest, as evidenced by the values of Observed_otus, Chao1, and Pielou_e (*p* < 0.05). In terms of the community diversity indices (Shannon and Simpson), the Pvi had the highest diversity, while the Tsi had the lowest diversity. In terms of good coverage, the average index of all samples was greater than 0.998, indicating a high sequencing depth.

### 3.5 Classification analysis of bacterial communities

**Figure 2:**
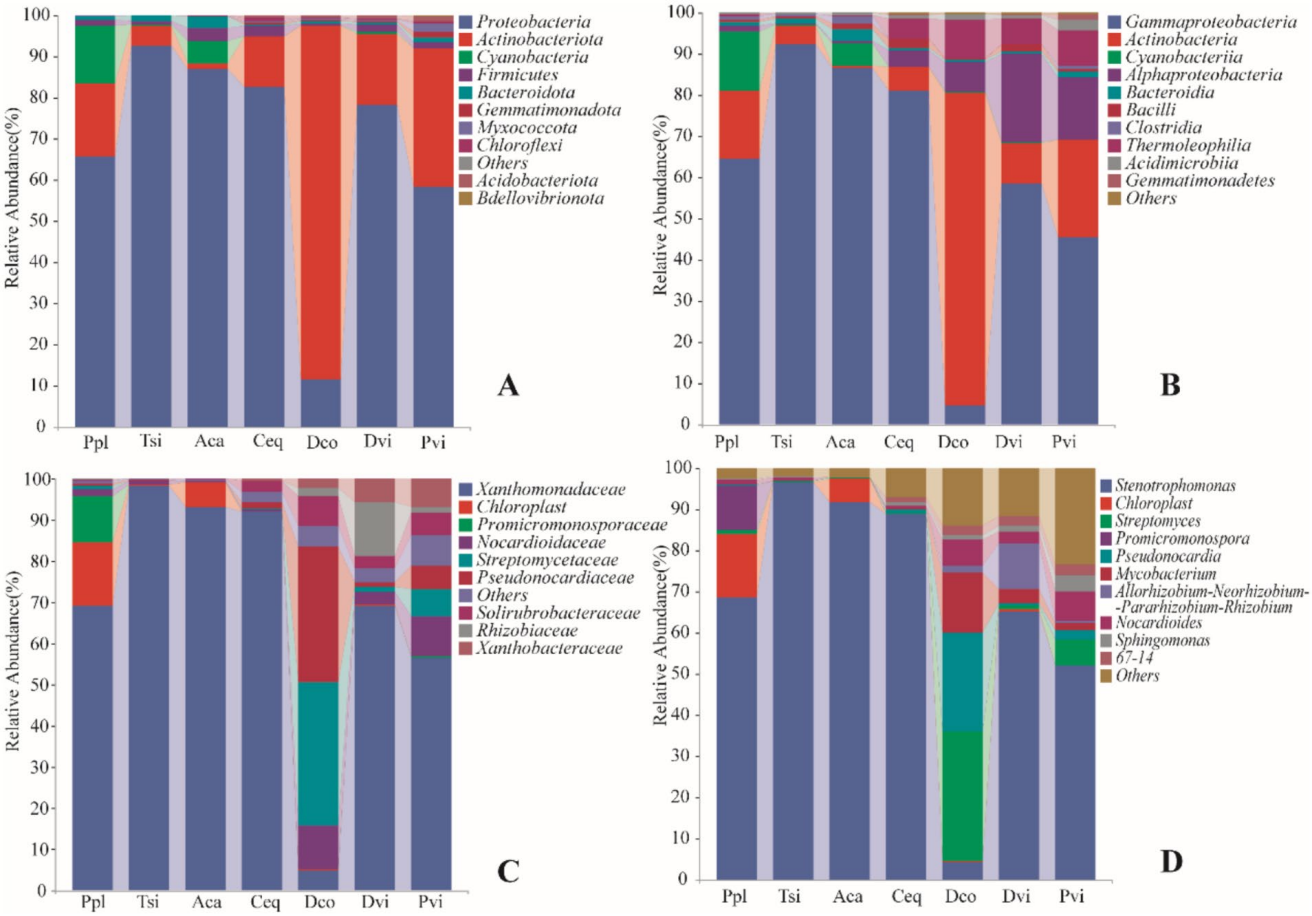
Relative abundance of soil bacteria in different samples at the phylum **(A)**, class **(B)**, family **(C)**, and **(D)** genus.

In all of the samples, a total of 28 phyla were identified. Figure 2A shows the changes in the abundance of the 10 most abundant phyla in the samples. *Proteobacteria* was the most abundant phylum in all of the samples, accounting for 67.89% of the total number of endophytic bacteria. *Proteobacteria* was the highest in the Tsi. The *Actinobacteria* were the second most abundant phylum in the samples, accounting for 24.65%. Making up 85.69% of the total bacteria present in the Dco, the *Actinobacteria* were also the most abundant. *Proteobacteria* was the second abundant phylum in the Dco sample, but its abundance was lower than that of the other six plant species (Dco is 9,214, Ppl is 52,519, Tsi is 73,862, Aca is 69,181, Ceq is 65,739, Dvi is 62,451, and Pvi is 46,366) (*p* < 0.05).

At the class level (Figure 2B), *Gammaproteobacteria* had the highest abundance (60.93%), followed by *Actinobacteria* (19.17%), *Alphaproteobacteria* (6.96%)*, and Cyanobacteriia* (2.89%). The abundance of *Gammaproteobacteria* in Tsi was greater than in Aca (73,475 and 68,743, respectively), and its abundance in Ceq was also greater than in Ppl (62,685 and 51,381, respectively) (*p* < 0.05). The abundance of *Alphaproteobacteria* in Dvi exceeded that in Pvi (16,682 and 11,634, respectively) (*p* < 0.05), and the abundance of the Ceq sample was significantly lower than that in Dco (3,045 and 5,553, respectively) (*p* < 0.05).

At the family level (Figure 2C), *Xanthomonadaceae*, *Chloroplast* and *Promicromonosporaceae* were the three families with the greatest abundance. The abundance of *Xanthomonadaceae* in Tsi rhizosphere soil was significantly greater than that in Aca rhizosphere soil. The Tsi abundance was 72,557, while the Aca abundance was 67,751. Additionally, Pvi showed a significant decreasing trend compared to Dvi, with Pvi and Dvi abundances of 29,673 and 42,742, respectively (*p* < 0.05). The *Streptomycetaceae* family was the most abundant in Dco at 20,326, while Dvi was slightly more abundant than Ppl (577 and 543, respectively) (*p* < 0.05).

At the genus level (Figure 2D), *Stenotrophomonas* was the most prevalent genus across all the samples, followed by *Chloroplast Streptomyces*, *Pseudonocardia*, and *Promicromonospora*. The abundance of *Stenotrophomonas* in the endophytic bacterial communities of the samples showed the following trend: Tsi > Aca > Ceq > Ppl > Dvi > Pvi > Dco (*p* < 0.05). For *Chloroplast* and *Pseudonocardia*, the abundances in the remaining five samples were very low, except for Ppl and Aca (with abundances of 11,130, 8,063, and 4,286, respectively). The abundances in the remaining five samples were as follows: Tsi (176, 0), Ceq (0, 3), Dco (83, 54), Dvi (286, 0), and Pvi (0, 6) (*p* < 0.05).

### 3.6 Structural differentiation of microbial communities

**Figure 3:**
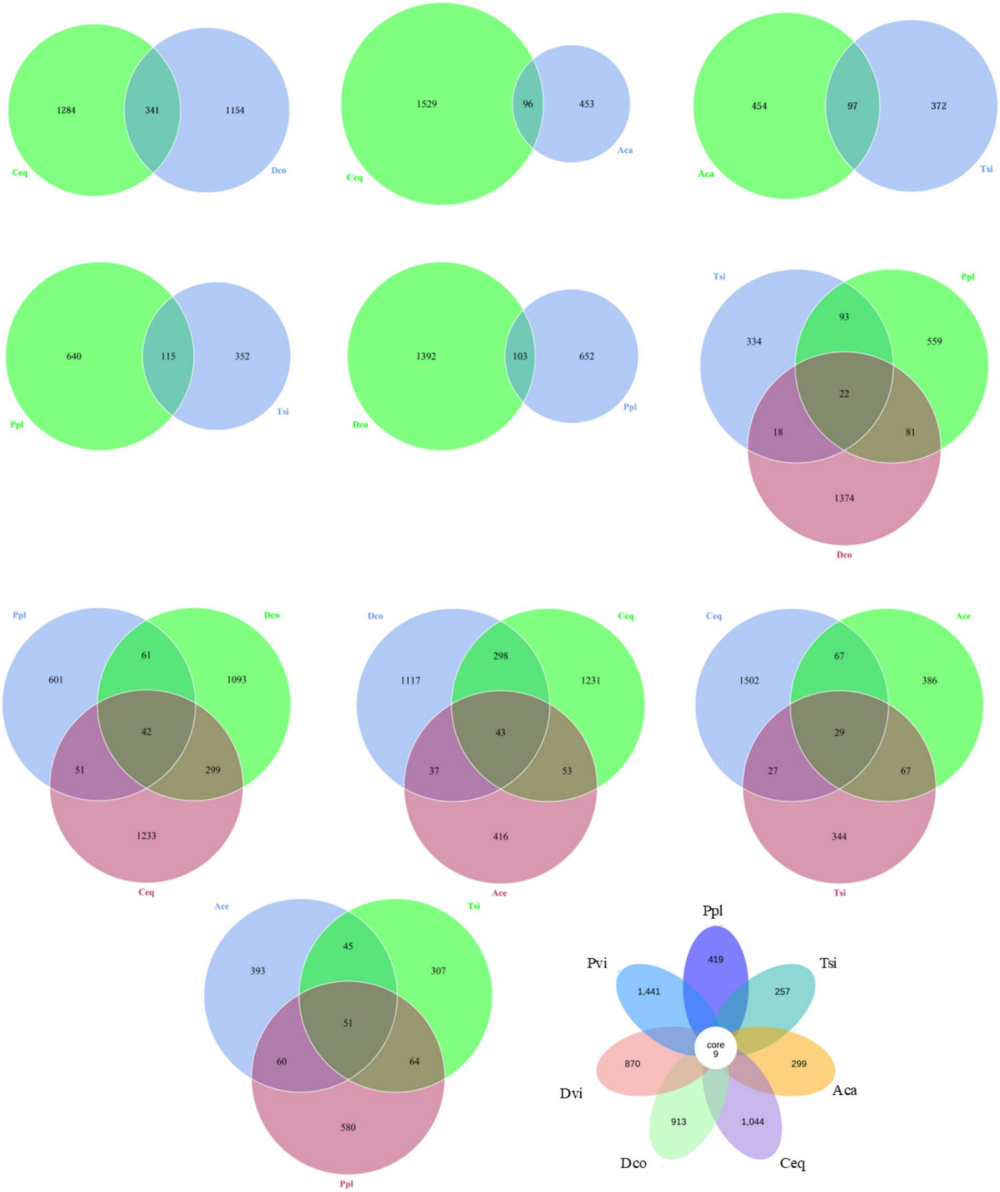
Shared and unique OTU analysis among different samples.

For specificity and consensus OTU analysis among different samples (shown in Figure 3), two adjacent samples were directly compared: Dco and Ceq. The former generated 1,154 specific OTUs, while the latter generated 1,284 specific OTUs. Compared to Aca and Ceq, Aca and Ceq generated 453 specific OTUs, while Ceq generated 1,529 specific OTUs. Compared to Aca, Tsi generated 372 specific OTUs, while Aca generated 454 specific OTUs. In comparison, Tsi generated 352 specific OTUs, while Ppl generated 640 specific OTUs. Compared to Ppl and Dco, Ppl generated 652 specific OTUs, while Dco generated 1,392 OTUs.

Comparison of three adjacent samples (as shown in Figure 3): Tsi, Ppl, and Dco. Tsi generated 334 specific OTUs, Ppl generated 559 specific OTUs, and Dco generated 1,374 specific OTUs. There were a total of 22 OTUs among the three groups. A comparison of Ppl, Dco and Ceq revealed that Ppl generated 601 specific OTUs, Dco generated 1,093 specific OTUs, and Ceq generated 1,233 specific OTUs, for a total of 42 OTUs among the three. A comparison of Dco, Ceq, and Aca revealed that Dco generated 1,117 specific OTUs, Ceq generated 1,231 specific OTUs, and Aca generated 416 specific OTUs. There were a total of 43 OTUs among the three. A comparison of Ceq, Aca and Tsi revealed that Ceq generated 1,502 specific OTUs, Aca generated 386 specific OTUs, and Tsi generated 344 specific OTUs, with a total of 29 OTUs among the three. In comparison, Aca generated 393 specific OTUs, Tsi generated 307 specific OTUs, and Ppl generated 580 specific OTUs, for a total of 51 OTUs among the three. In total, all of the samples had nine core OTUs (*Stenotrophomonas, Chryseobacterium, Lactobacillus, Clostridium_sensu_strictto_12, Prevotella, Lactobacillus, Bradyrhizobium, Nocardioides*, and Delftia). Each sample contained 257 to 1,441 specific OTUs (shown in Figure 3).

**Figure 4:**
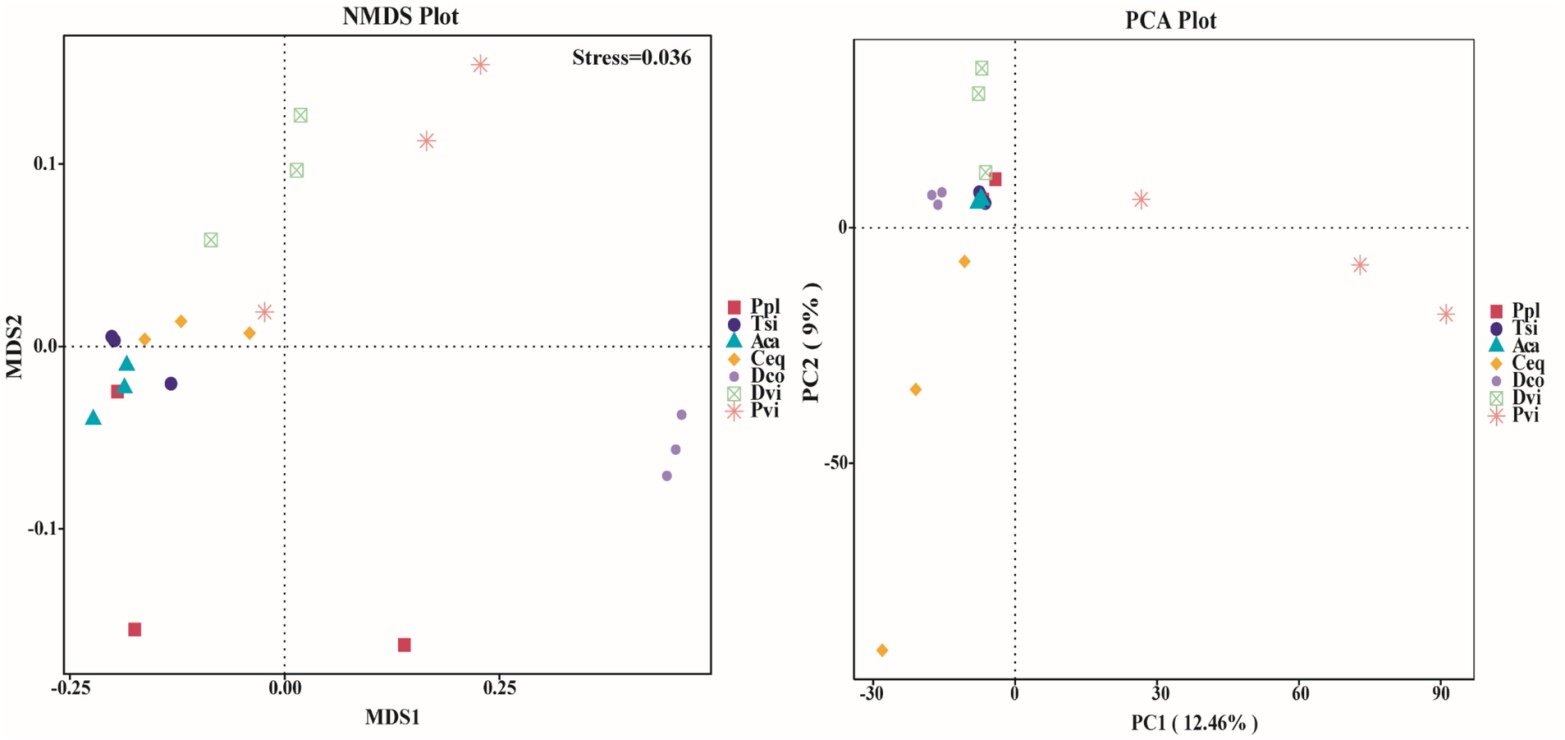
Beta diversity between different samples based on NMDS and PCoA.

We employed NMDS and PCA to evaluate the variations in the endophytic bacterial communities within the roots of the various samples (Figure 4). We found that most species presented a high level of similarity. Since the stress value was less than 0.05 in the NMDS analysis, it can be concluded that the results were highly representative. The microbiomes of five species, Aca, Tsi, Ceq, Ppl, and Pvi, overlapped. Notably, the three species Tsi, Ceq, and Aca were more closely related and presented greater similarity than Dvi and Dco. PCA revealed that in the PC1 dimension, with the exception of Pvi, the projection distances of the other six species were relatively close. Therefore, the species richness values of Ceq, Aca, Tsi, Ceq, Ppl and Dco were more similar. In addition, in the PC2 dimension, the projection distances of Dco, Aca, Tsi, and Ppl were the shortest, suggesting that the richness of these four species was the most similar.

### 3.7 Metabolic functions of the endophytic bacterial communities

**Figure 5:**
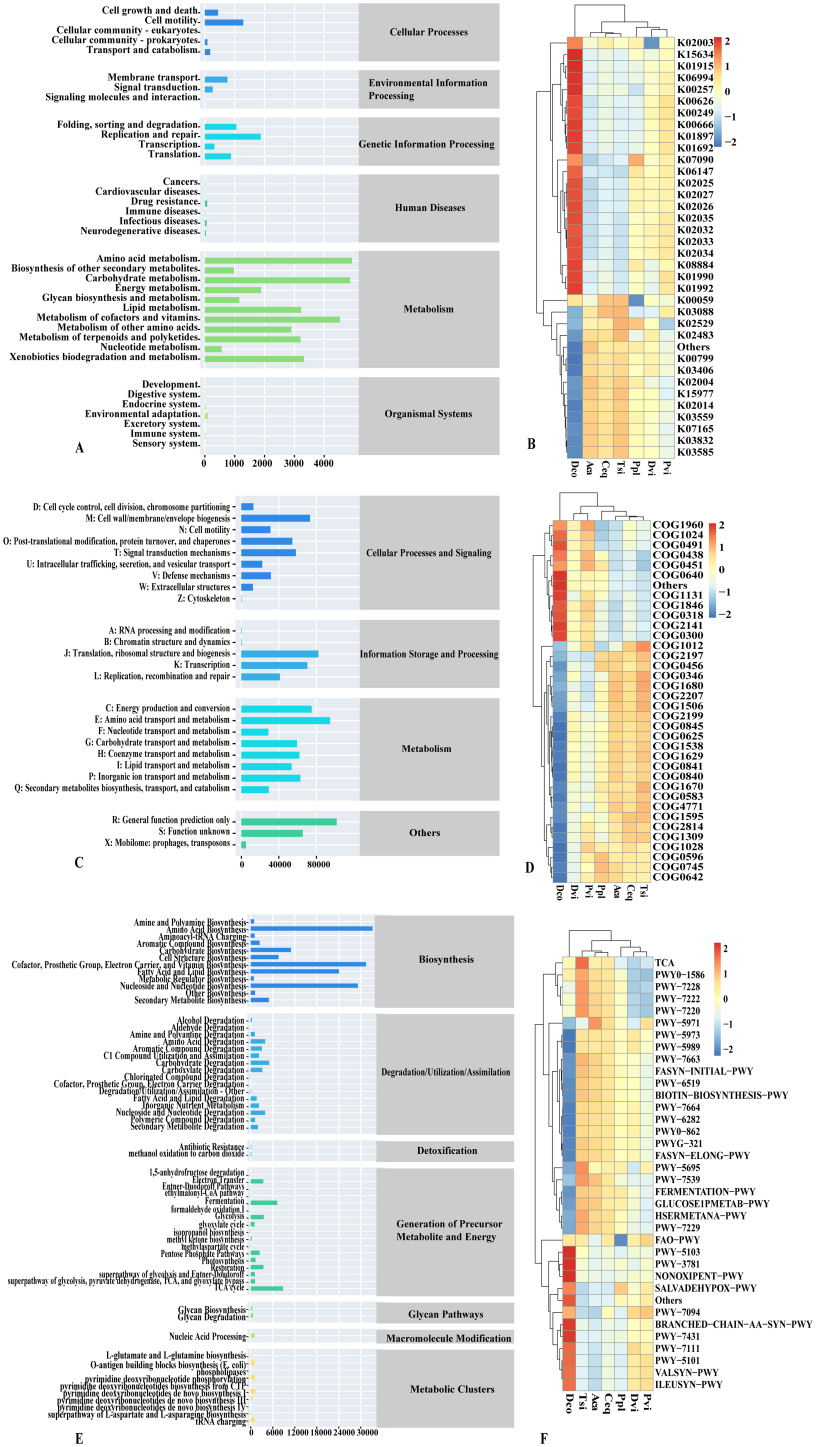
PICRUSt2 function prediction **(A,B)** KEGG, **(C,D)** COG, **(E,F)** MetaCycE.

We employed PICRUSt2 to forecast the precise functions of the bacteria found in the samples. Using the KEGG database, bacterial genes were categorized into six different groups (Figure 5A): metabolism, genetic information processing, environmental information processing, and cellular processes, Organismal Tsistems and Human Diseases. Metabolism was the most abundant KEGG pathway in all of the samples (80.75%), followed by genetic information processing and cellular processes, accounting for 10.55 and 5.12%, respectively. The organic system had the lowest proportion of genes, at 0.44%. At the second level, amino acid metabolism, carbohydrate metabolism, cofactors, and vitamin metabolism were the most abundant KEGG metabolic functions, accounting for 12.62, 12.46, and 11.58%, respectively.

In response to the functional differences among different species, we selected the top 35 functions to draw a heatmap for analysis (Figure 5B). We found that the functional differences among the species were significant. Compared with those in the other six samples, the expression levels of genes associated with the following functions were significantly increased in the Dco group: glnA, GLUL; glutamine synthetase (K01915); acetyl-CoA C-acetyltransferase (K00626); fatty-acyl-CoA synthase (K00666); long-chain acyl-CoA synthetase (K01897); enoyl-CoA hydratase (K01692); ABCB-BAC; ATP-binding cassette, subfamily B; bacterial (K06147); and ABC.MS. P; multiple sugar transport system permease protein (K02025), ABC.MS. S; multiple sugar transport system substrate-binding protein (K02027), ABC.MS. the P1; multiple sugar transport system permease protein (K02026), ABC.PE. S; peptide/nickel transport system substrate-binding protein (K02035), ABC.PE. A1; peptide/nickel transport system ATP-binding protein (K02032), ABC.PE. P peptide/nickel transport system permease protein (K02033), ABC.PE. the P1; peptide/nickel transport system permease protein (K02034), serine/threonine protein kinase, bacterial (K08884), ABC-2. A; ABC-2 type transport system ATP-binding protein (K01990), ABC-2. P; ABC-2 type transport system permease protein (K01992), GST, gst; glutathione S-transferase (K00799); in addition, the Dco group was in exbD; biopolymer transport protein ExbD (K03559), fecR; transmembrane sensor (K LacI family transcriptional regulator (K02529)), mcp; methyl-accepting chemotaxis protein (K03406), acrA, mexA, adeI, smeD, mtrC, cmeA; membrane fusion protein, multidrug efflux system (K035E85); rpo; RNA polymerase sigma-70 factor, ECF subfamily (K03088), two-component system, OmpR family, response regulator (K02483), GST, gst; glutathione S-transferase (K00799), mcp; methyl-accepting chemotaxis protein (K03406); ABC.CD. P; putative ABC transport system permease protein (K02004), putative oxidoreductase (K15977), TC.FEV. The expression of OM, iron complex outer membrane receptor protein (K02014), fecR, transmembrane sensor (K07165), and putative long-chain acyl-CoA synthase (K03822) was significantly lower than that in the other groups. The expression of Aca, Ceq, and Tsi contrasted with that of the Dco group. The expression of the Dco group decreased, while the expression of the Aca, Ceq, and Tsi groups increased.

The COG database (Figure 5C) can be divided into three categories, with metabolism accounting for 41.26%, followed by information storage and processing, and cellular processes and signal transduction, accounting for 26.25 and 17.25%, respectively. Secondary COG metabolism analysis revealed that amino acid transport and metabolism, translation, ribosome structure and biogenesis, and energy production and conversion, as well as cell wall/membrane/envelope biogenesis, accounted for the greatest percentage of patients, making up 8.39, 7.28, 6.65, and 6.49%, respectively. Similarly, we performed intergroup comparisons using a heatmap (Figure 5D). Dco was compared with other groups in terms of the levels of Acyl-CoA dehydrogenase related to the alkylation response protein AidB (COG1960), enoyl-CoA hydratase/carnitine racemase (COG1024), DNA repair exonuclease SbcCD ATPase subunit (COG0419), glycosyltransferase involved in cell wall bisynthesis (COG0438), nucleoside-diphosphate-sugar epimerase (COG0451), DNA-binding transcriptional regulator, ArsultR family (COG0640, ABC-type Atransgase) component (COG1131), DNA-binding transcriptional regulator, MarR family (COG1846), Acyl-CoA synthetase (AMP-forming)/AMP-acid ligase II (COG0318), Flavin-dependent oxidoreductase, luciferase family (COG2141), and short-chain. The functional expression of dehydrogenase (COG0300) was upregulated, while other selected functions were downregulated. The expression of functions in the Aca, Ceq, and Tsi groups contrasts with that in the Dco group. The functions upregulated in the Dco group were downregulated in the Aca, Ceq, and Tsi groups.

The MetaCyc database (Figure 5E) categorizes bacterial genes into seven different groups:Biosynthesis was the predominant category in the samples, making up 67.18% of the total count, followed by generation, degradation/utilization/assimilation of detoxification precursor metabolites and energy, accounting for 15.53 and 13.64%, respectively. In the second stage, amino acid biosynthesis (15.09%) accounted for the largest proportion, followed by cofactor, prosthetic group, electron carrier, and vitamin biosynthesis (14.28%), and nucleoside and nucleotide biosynthesis (13.27%). The biosynthesis of fatty acids and lipids (10.93%), carbohydrate biosynthesis (4.99%), and the TCA cycle (4.01%) were investigated. A comparison of the differences between the various test groups was performed (Figure 5F). A comparison of the Tsi group, the Aca group, and the Ceq group revealed that the functional expression was generally consistent, which was somewhat consistent with the opposite functional expression of Dco: Dco in palmitate biosynthesis II (bacteria and plants) (PWY-5971), cis-vaccenate biosynthesis (PWY-5973), stearate biosynthesis II (bacteria and plants) (PWY-5989), gondoate biosynthesis (anaerobic) (PWY-7663), superpathway of fatty acid biosynthesis initiation (FASYN-INITIAL-PWY), 8-amino-7-oxononanoate biosynthesis I (PWY-6519), biotin biosynthesis I (BIOTIN-BIOSYNTHESIS-PWY), oleate biosynthesis IV (anaerobic) (PWY-7664) (PWY-6282), (5Z)-dodec-5-enoate biosynthesis (PWY0-862), mycolate biosynthesis I (from (5Z)-dodec-5-enoate) (PWY-6282), (5Z)-dodec-5-enoate biosynthesis (PWY0-862), mycolate biosynthesis (PWYG-321), fatty acid elongation - saturated (FASYN-ELONG-PWY), urate biosynthesis/inosine 5′-phosphate degradation (PWY-569), 6-hydroxymethyl-dihydropropterin diphosphate biosynthesis III (Chlamydia) (PWY-7539), mixed acid fermentation (FERMENTATION-PWY), glucose and glucose-1-phosphate degradation (GLUCOSE1PMETAB-PWY), L-methionine biosynthesis III (HSERMETANA-PWY), superpathway of adenosine nucleotides *de novo* biosynthesis I (PWY-7229) showed down-regulation of their functional expression. A comparison of the Dco group and others revealed that L-isoleucine biosynthesis III (PWY-5103), aerobic respiration I (cytochrome c) (PWY-3781), the pentose phosphate pathway (nonoxidative branch) (NONOXIPENT-PWY), adenosine nucleic acid degradation II (SALVADEHYPOX-PWY), fatty acid salvage (PWY-7094), the superpathway of branched amino acid biosynthesis (BRANCHED-CHAIN-AA-SYN-PWY), aromatic biogenic amine degradation (bacteria) (PWY-7431), pyruvate fermentation to isobutanol, and the functional expression of (engineered) (PWY-7111), L-isoleucine biosynthesis II (PWY-5101), L-valine biosynthesis (VALSYN-PWY), and L-isoleucine biosynthesis I (from threonine) (ILEUSYN-PWY) were upregulated.

### 3.8 Differentiation of the metabolic function of the microbial community

PCA was used to assess the variations in bacterial function across the different samples (Supplementary Figure S2). To a certain extent, the functional differentiation of rhizosphere microbes among different species in mining areas has occurred. Aca had the highest degree of dispersion, Ppl had the lowest, and Dco was the farthest from the rest of the samples, indicating greater functional differences.

### 3.9 Correlation analysis

**Figure 6:**
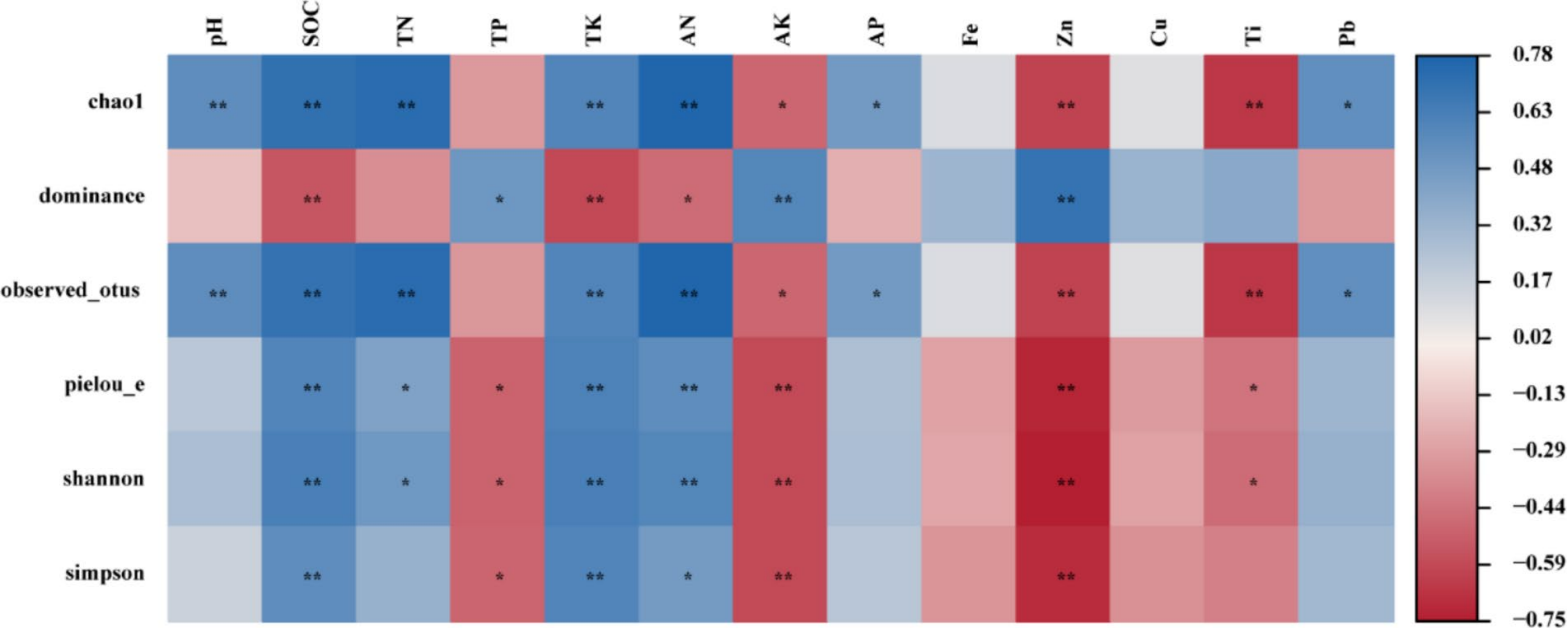
Pearson correlation analysis.

Pearson correlation analysis (PC, *p* < 0.05) was used to evaluate the correlations between the soil physicochemical properties and the Alpha diversity indices of the endophytic bacteria (Figure 6). The results revealed that the SOC, TK, AN, AK, and Zn contents were significantly correlated with alpha diversity. A strong positive correlation was observed between pH and Chao1. Furthermore, a strong positive correlation was noted between the SOC content and Chao1, Observed_otus, Pielou’s evenness, Shannon, and Simpson. In contrast, the AK, Zn, and Ti contents were significantly negatively correlated (*p* < 0.05) with Chao1, Observed_otus, Pielou’s evenness and Shannon.

**Figure 7:**
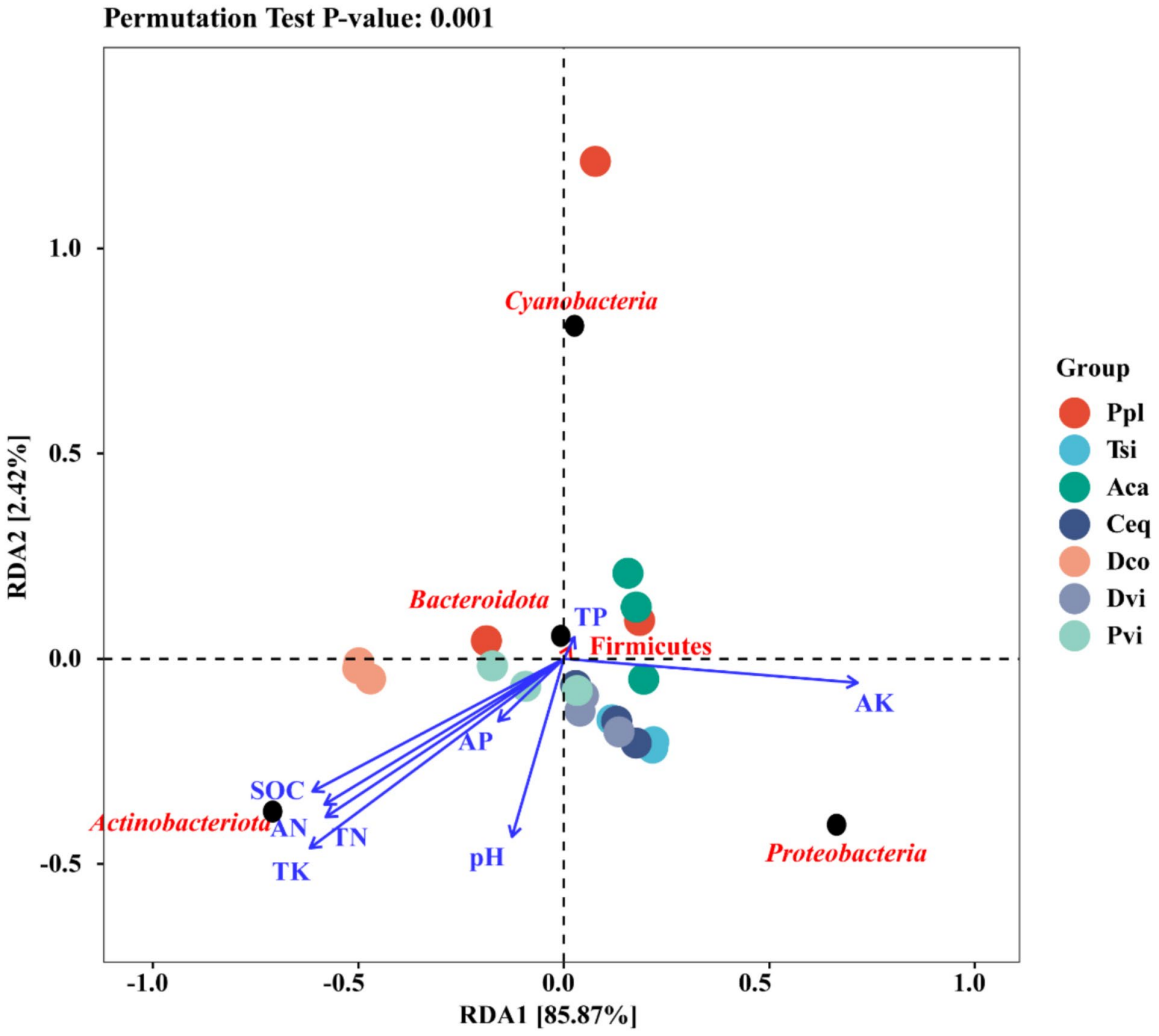
Redundancy analysis.

To further investigate the effects of environmental factors on the endophytic bacterial microflora of the rhizosphere soil and roots, redundancy analysis (RDA) was used to focus on the top 10 microbial species at the phylum level and their relationships with soil environmental factors (Figure 7). RDA1 and RDA2 explained 85.87 and 2.42%, respectively, of the total variance observed by the analyzed species, significantly explaining the complex relationship between environmental factors and the composition of microflora. Among them, *Actinobacteria* were significantly positively correlated with the contents of SOD, AN, TN, and TK in the soil. Moreover, *Bacteroidota* and *Firmicutes* exhibited a significant positive correlation with the TP content in the soil.

## 4 Discussion

### 4.1 Effects of ilmenite ecological restoration on the surrounding soil elements

In this study, an ilmenite ore area was taken as the research object to determine the pH, SOC, TN, and TK of the soil in the area. pH is an important factor affecting the soil microbiome (Xiao et al., 2021). The tests revealed that the pH values of Ppl and Dvi were lower than those of CK, which may be due to soil degradation caused by mining disturbance, resulting in a decrease in soil pH. The pH values of Tsi, Aca, Ceq, Dco, and Pvi were greater than those of CK. It is possible that in an oxidative environment, Fe(II) and Fe(III) in the soil readily form hydroxides, thus increasing the pH value (Huang et al., 2019). SOC plays a dual role in plants, serving as both a substrate and an energy source for various physiological and biochemical processes, thereby indicating the soil fertility level to some extent. The SOC content in the experimental group was lower than that in the control group, and the accumulation of Ceq in the experimental group was the highest. This is likely because heavy metals significantly inhibit the carbon utilization ability and enzyme activity of plants and microorganisms (Li et al., 2011). A high content of organic matter can promote the metabolism of soil microbes to produce enzymes (Xu et al., 2024), resulting in a decrease in soil fertility. Nitrogen and phosphorus play crucial roles in organisms and serve as significant constraints on soil productivity, impacting soil physicochemical properties (Fan et al., 2021). In terms of TN content, Ceq, Dco, and Pvi were significantly greater than those in CK, indicating that the specific conditions of the mining area promoted the uptake of nitrogen by these two types of plants. In addition, the AN content of all the plants was lower than the CK level, indicating once again that mining activities limit the uptake of nitrogen by plants. This is because the AN content depends on the content and maturity of soil organic matter (SOM), and a decrease in organic matter inevitably leads to an increase in nitrogen content. Phosphorus plays a crucial role in maintaining soil fertility. The soil’s phosphorus pool is a crucial source of many elements necessary for plant growth and development, providing most of the phosphorus plants need (Billah et al., 2019). The AP contents of the seven plants were lower than those of the CK plants. It is also related to soil organic matter. The SOM is the most conducive factor for maintaining phosphorus in the soil (Silva et al., 2016). The reduction in organic matter will also be reduced to a certain extent. Soil AK is easily taken up and used by plants, making it an important indicator for characterizing the potassium supply level in soil. The AK content was consistent with that of AP and lower than that of CK. There were significant spatial variations in the AK content, which were influenced by soil pH, soil water levels, soil texture, and type of clay mineral (Li et al., 2021). In addition to Ceq (Qin et al., 2024) and Pvi (Salas-Luévano et al., 2017), existing methods have been used for heavy metal control, and the remaining five plant species have been used for the first time in the ecological restoration of mining areas. Therefore, Ceq and Pvi have high feasibility and can be further promoted.

### 4.2 Effects of the ecological restoration of ilmenite on the surrounding microbial communities

The microbial composition differs greatly in the process of ilmenite ecological restoration, which affects the community structure (Liu et al., 2023). In contrast to alpha diversity, when the indices were compared, we found significant differences among the seven plants. In terms of the Chao1 and Shannon, Pvi, Dco, and Dvi had relatively high values, indicating that, to a certain extent, the species richness and community diversity of these three plants were promoted in the mining area environment. Under heavy metal pollution, plants adapt to environmental changes by influencing the assembly of bacterial communities, which in turn affects the composition and diversity of these communities (Jiang et al., 2019; Zhang et al., 2015). Further analysis of the Simpson revealed that Dco was significantly increased. This indicates that in this environment, the distribution of species in the community is more uniform, and the diversity is better maintained. However, there was no significant difference between Ppl and the other methods in terms of the Simpson. Therefore, Ppl may be sensitive to or have a low response to environmental changes in the mining area. In addition, among all the indicators, Pvi presented the highest abundance, which to some extent indicates the strong adaptability and biological maintenance ability of Pvi plants in the mining area environment.

In addition, further studies on the factors affecting the diversity of microbial communities were conducted through Pearson correlation analysis. This analysis was based on the alpha diversity, heavy metal content, and soil properties. Chao1 and observed_otus had significant positive correlations with pH, SOC, TN, and TK and significant negative correlations with Zn and Ti. This finding once again indicates that the abundance of the bacterial community is primarily affected by soil properties (Yang et al., 2023). However, there were significant correlations between the Shannon index and the Simpson index, as well as between the SOC and Zn. Therefore, the diversity of the endophytic bacterial community is affected by both soil characteristics and heavy metal pollution. Jiang et al. (2019) found that the key environmental variable for the composition and diversity of soil bacterial communities was soil characteristics, which is consistent with our findings. The solubility of heavy metals changes under the influence of soil pH, which in turn affects the bioavailability of heavy metals, resulting in changes in their toxicity (Awasthi et al., 2016).

Redundancy analysis revealed that *Actinobacteriota* was significantly positively correlated with the contents of SOD, AN, TN, and TK in the soil. This result was consistent with those of previous studies (Pham et al., 2024). *Actinobacteriota* became the dominant species in this study, with a greater proportion of *Actinobacteriota* in oilfield areas, farms, and cornfields than in soils in mining areas. It has been reported that *Actinobacteriota* contains heavy metal resistance genes (Yan et al., 2020). In this study, Fe, Zn, Cu, Ti, and Pb were found in the soil in the mining area, indicating that the Actinobacteria may be resistant to these metals. *Proteobacteria, Firmicutes* are also the main metal-resistant bacteria (Yan et al., 2020), based on the results and existing studies, we believe that metal tolerance in drug-resistant bacteria is achieved through the export of metals by the corresponding transport proteins through pumps and ion channels, or reduction by redox reactions. Bacterial metal tolerance may be related to DNA repair capacity. Microbial DNA can be damaged and produce oxidative stress under heavy metal stress, and DNA recombination can repair damaged genes, and repair-associated genes have been shown to be present in microbial genomes and plasmids, so that these genes can be exchanged by gene transfer via plasmids or transposons (Nanda et al., 2019; Bruins et al., 2000).

In terms of functional predictions for endophytic bacteria, we identified four proteins related to peptide/nickel transport, three proteins related to ATP, two proteins related to ABC transport, and glutathione S-. The function of transferase was upregulated, indicating that it is related to the uptake and transport of heavy metals. This is different from what was reported by Liu et al. (2024b). The study results were consistent. Glutathione S-transferase (GST) is an important protein for the detoxification of organic xenobiotics and endophytic metabolites, and it is a key enzyme in the GSH binding reaction. The defense system against oxidative stress and other negative consequences increases GST activity when metals enter an organism (Işık et al., 2023). In this study, glutathione S-transferase was upregulated, indicating that in this environment, endophytic bacteria secrete more glutathione S-transferase to resist heavy metal damage and thus promote the growth of plants and microorganisms. The upregulated metabolites are critical for maintaining normal cellular metabolic stability. Metal transporter proteins, which are situated on the cell membrane, play a crucial role in the absorption and transportation of metal elements (Chen et al., 2017). ABC transporter proteins serve as powerful transporters (Wang et al., 2022) and can transfer inorganic ions, amino acids, and metal ions (Guo et al., 2022). In addition, ABC transporter proteins are essential for protecting against virulence factors and drugs, as well as for regulating metal ion levels by moving them across the cell membrane in various forms (Do et al., 2018). The function of acyl-CoA-related enzymes is upregulated. It can be inferred that the activity of the donor acyl-CoA is increased to promote phytorepair and the removal of harmful heavy metals from the environment (Xiao and Chye, 2011). Whether the above process occurs is to be verified by subsequent experiments.

In summary, Pvi, Dco, and Dvi showed high abundance levels in heavy metal environments, as well as strong environmental adaptability. The two bacteria, *Actinobacteria* and *Firmicutes*, are dominant bacteria that can be made into microbial agents. These agents are expected to be used in the future for ecological remediation of heavy metal environments such as mines and ironworks.

## 5 Conclusion and future perspectives

This study investigated the ecological restoration of abandoned ilmenite mines by seven common plants. The results revealed that the abandonment of ilmenite significantly increased the contents of total phosphorus, total potassium, available potassium, iron, and lead in the surrounding soils. It also affected the richness and diversity of endophytic bacterial communities. Pvi had the highest richness, while Tsi had the lowest richness (*p* < 0.05). A total of 28 phylums, 69 classs, 171 orders, and 521 genus were identified; the nine core OTUs shared. Beta diversity analysis revealed that the community structure of the endophytic bacteria differed during the remediation process at the ilmenite site. Functional prediction revealed upregulation of Dco transporter protein function, DNA-binding transcriptional regulators, glyoxalase or related metal-dependent hydrolases, acyl coenzyme A synthetases, ATPase components, amino acid synthesis, and cellular respiration-related functions. Pearson correlation analysis revealed that the SOC, TK, AN, AK, and Zn contents were significantly correlated with α diversity. Redundancy analysis (RDA) revealed that *Actinobacteriota* was significantly positively correlated with soil SOD, AN, TN, and TK contents. For the first time, this study revealed the interactions among plants, endophytic bacteria and soil pollutants, laying a theoretical basis for screening specific plant endophytic bacteria for ecological restoration.

In this study, we screened plants with strong growth ability and endophytic bacteria with high abundance in heavy metal environments to demonstrate their adaptive abilities to heavy metals. This will lay a theoretical foundation for the ecological restoration of mine sites. In order to prove its ability of ecological restoration, the growth of plants and the differentiation of endophytic bacteria should be continuously monitored in the later stage. In addition, the functions and mechanisms of *Actinobacteriota* and *Firmicutes* in metal detoxification and soil restoration need to be further investigated.

## Data Availability

The original contributions presented in the study are publicly available. This data can be found here: https://www.ncbi.nlm.nih.gov/sra/PRJNA1230397.
