# Supplementary material for: Differentiation and response mechanisms of the endophytic flora of plants ecologically restored in the ilmenite area
Source: Front Microbiol. 2025 Mar 7;16:1555309. doi: 10.3389/fmicb.2025.1555309 (PMC11926159; doi:10.3389/fmicb.2025.1555309)
Supplement: Supplementary file 1 [file Data_Sheet_1.docx]

**Differentiation and response mechanisms of the endophytic flora of plants ecologically restored in the ilmenite area**

Xin Yu^a^, Junqiang Xu^b^, Ziping Zou^a^, Yunfeng Zhang^a^, Peng Wu^b^, Qiang Li^a*^

a Key Laboratory of Coarse Cereal Processing, Ministry of Agriculture and Rural Affairs, Sichuan Engineering & Technology Research Center of Coarse Cereal Industrialization, School of Food and Biological Engineering, Chengdu University, Chengdu 610106, China;

b Yunnan Plateau Characteristic Agricultural Industry Research Institute, Yunnan Agricultural University, Kunming, Yunnan 650500, China;

#Xin Yu and Junqiang Xu contribute equally to this work.

*Corresponding author: Qiang Li (liqiang02@cdu.edu.cn)

Phone: +86-028-84616653; Tel: +86-15196619794

*Present address: No. 2025, Chengluo Avenue, Longquanyi District, Chengdu 610106, Sichuan, PR China

.
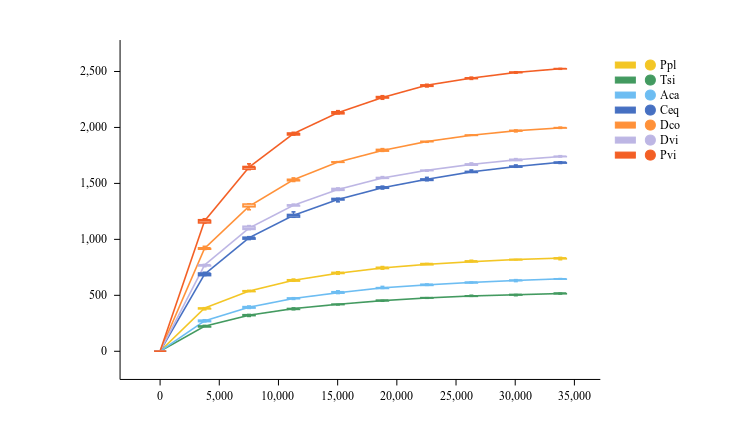


Fig. S1 Trends of observed OTUs in different samples changing with sequencing reads.


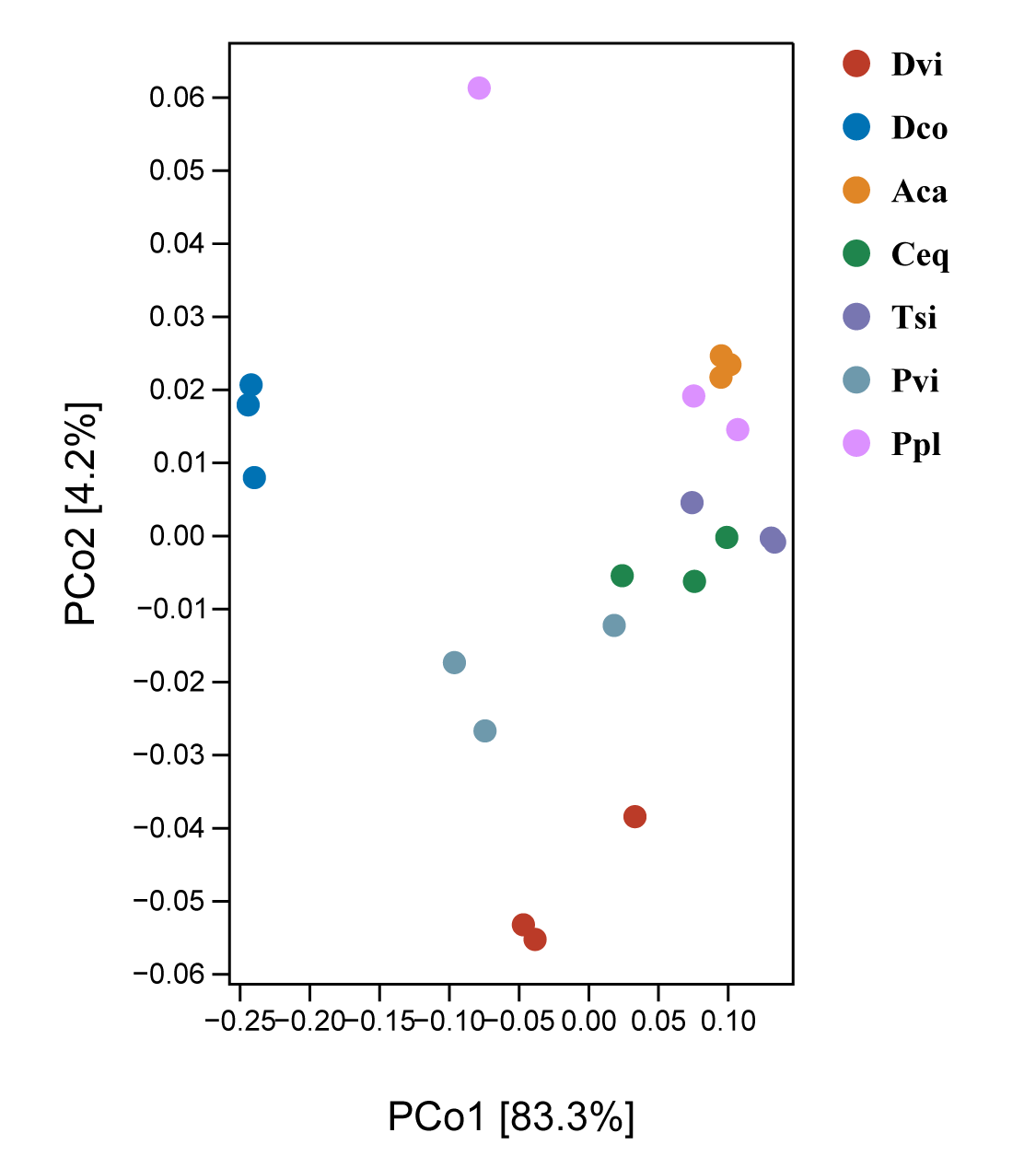


Fig. S2 Functional PCA of endophytic bacteria in different samples of plants Prediction

Table S1 Plant names, abbreviations, and sampling locations

| Plant Name | Abridgement | Sampling Location |
| --- | --- | --- |
| *Polygonum plebeium* R. Br. | Ppl | An abandoned mine in Wuding County, Chuxiong Yi Autonomous Prefecture, Yunnan Province (within 3 kilometers of the ilmenite area) |
| *Tournefortia sibirica* L. | Tsi | An abandoned mine in Wuding County, Chuxiong Yi Autonomous Prefecture, Yunnan Province (within 3 kilometers of the ilmenite area) |
| *Alhagi camelorum* Fisch. | Aca | An abandoned mine in Wuding County, Chuxiong Yi Autonomous Prefecture, Yunnan Province (within 3 kilometers of the ilmenite area) |
| *Casuarina equisetifolia* L. | Ceq | An abandoned mine in Wuding County, Chuxiong Yi Autonomous Prefecture, Yunnan Province (within 3 kilometers of the ilmenite area) |
| *Dryopteris coreano-montana* Nakai | Dco | An abandoned mine in Wuding County, Chuxiong Yi Autonomous Prefecture, Yunnan Province (within 3 kilometers of the ilmenite area) |
| *Dodonaea viscosa* Jacquem. | Dvi | An abandoned mine in Wuding County, Chuxiong Yi Autonomous Prefecture, Yunnan Province (within 3 kilometers of the ilmenite area) |
| *Pteris vittata* L. | Pvi | An abandoned mine in Wuding County, Chuxiong Yi Autonomous Prefecture, Yunnan Province (within 3 kilometers of the ilmenite area) |
